# Avacopan in Patients With Rapidly Progressive Glomerulonephritis Requiring Dialysis

**DOI:** 10.1016/j.ekir.2023.05.017

**Published:** 2023-05-29

**Authors:** Frank B. Cortazar, Jorge Cerda, Rahim Dhanani, Joseph Roglieri, Dominick Santoriello

**Affiliations:** 1 New York Nephrology Vasculitis and Glomerular Center, Albany, New York, USA; 2 Division of Nephrology, St. Peter’s Hospital, Albany, New York, USA; 3 Department of Pathology, Columbia University Irving Medical Center, New York, New York, USA

## Introduction

Antineutrophil cytoplasmic antibody (ANCA)-associated vasculitis (AAV) is a small-vessel vasculitis with a predilection for the kidney.^1^ Morbidity and mortality among patients with AAV are largely driven by kidney-related outcomes.^2^ In particular, the requirement for kidney replacement therapy (KRT) is associated with a marked decrease in patient survival and decreased quality of life.^3^ Therefore, therapies that can rapidly and effectively treat ANCA-associated glomerulonephritis (GN) have the potential to significantly improve patient outcomes.

Current standard-of-care therapy for severe AAV is glucocorticoids combined with either rituximab or cyclophosphamide.^4^ The addition of plasma exchange can be considered in patients with severe kidney involvement, particularly among those approaching the need for KRT.S1 Avacopan, an oral C5a receptor antagonist, was recently approved as an adjunctive therapy for AAV. In phase 3 ADVOCATE trial, patients with AAV randomized to avacopan had a noninferior rate of remission at 26 weeks and superior rate of sustained remission at 52 weeks compared with patients randomized to standard-of-care prednisone.^5^ Among the 81% of the study population with kidney involvement, patients treated with avacopan had more rapid reductions in albuminuria and greater recovery of estimated glomerular filtration rate (eGFR) at 26 and 52 weeks. Notably, patients with an eGFR <15 ml/min per 1.73 m^2^, including patients requiring KRT, were excluded from the ADVOCATE trial and there is currently limited data available on using avacopan in this important subgroup. Standard practice at our center has evolved to use avacopan as a component of first-line induction therapy, in combination with cyclophosphamide or rituximab and a rapid glucocorticoid taper, for patients with severe AAV. Here, we present 3 consecutive patients with *de novo* ANCA GN who were treated with avacopan following initiation of hemodialysis.

## Case Presentation

### Case 1

**Figure 1:**
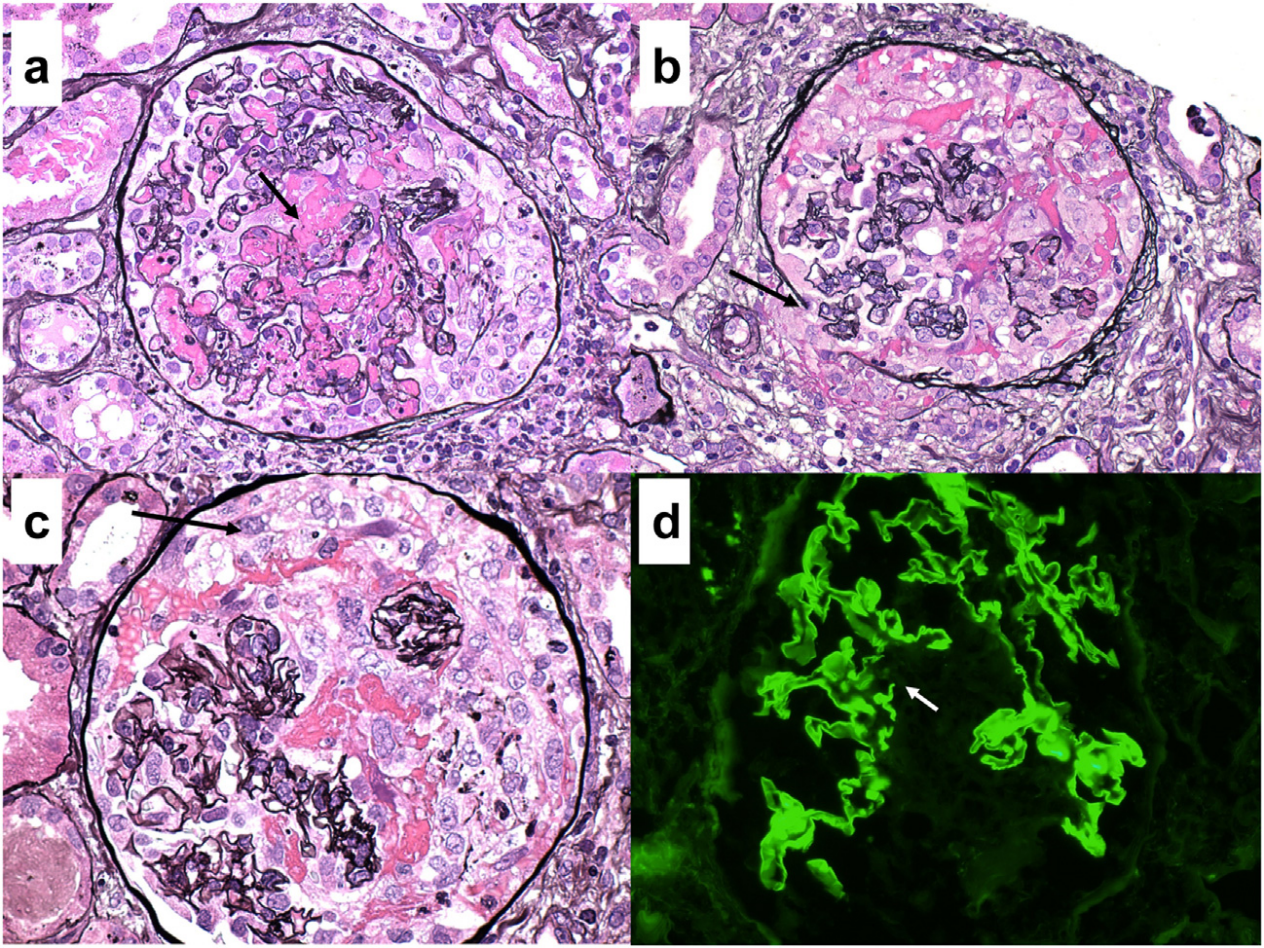
(a) A representative glomerulus from case #1 exhibits segmental GBM glomerular basement membrane rupture (arrow) associated with fibrin extravasation into the urinary space and segmental cellular crescent formation. Marinating neutrophils, some of which are undergoing karyorrhexsis, are admixed with fibrin in several capillary loops (×200, Jones methenamine silver; JMS). (b) A representative glomerulus from case #2 displays a circumferential cellular crescent compressing the underlying glomerular tuft. There is also focal disruption of Bowman’s capsule (×200, JMS). (c) A segmental cellular crescent (arrow) from case #3’s kidney biopsy. The uninvolved portions of the glomerular tuft are normocellular with patent capillary lumina, typical of ANCA-mediated glomerulonephritis (×200, JMS). (d) Immunofluorescence staining for case #3 revealed intense (3+) linear staining of glomerular basement membranes for IgG, typical of anti- glomerular basement membrane- GBM disease. Foci of glomerular basement membrane GBM rupture were apparent (arrow). This patient was also found to have proteinase-3 PR3-ANCA seropositivity (×200, immunofluorescence microscopy).

An 89-year-old woman with a history of hypertension, hypothyroidism, and hyperlipidemia presented to the hospital with weight loss, malaise, and acute kidney injury. Baseline creatinine 2 months prior was 0.76 mg/dl and had risen to 2.3 mg/dl on presentation with an active urinary sediment. Myeloperoxidase-ANCA was elevated at 417 units (reference <1). Renal biopsy revealed pauci-immune necrotizing and crescentic GN (mixed class)S2 with cellular crescents involving 11 of 16 viable glomeruli (Figure 1a). Global glomerulosclerosis was present in 45% of glomeruli, and interstitial fibrosis and tubular atrophy involved 30% to 40% of the renal cortex.

The patient was treated with methylprednisolone 500 mg i.v. daily for 3 days followed by a prednisone taper, rituximab 1000 mg × 2, and plasma exchange (5 sessions). Hemodialysis was initiated on hospital day 10 because of uremia and volume overload (Table 1). Avacopan 30 mg twice daily was started on hospital day 15. Prednisone was tapered as outlined in Supplementary Table S1. The patient discontinued dialysis on day 125 and remains in clinical remission with a serum creatinine of 2.5 mg/dl after 265 days of follow-up (Table 1). Maintenance therapy with rituximab was initiated 6 months after presentation and the patient remains on avacopan.

**Table 1:** Clinical course of patients treated with avacopan

Patient	Cr at presentation (Day 0)	Cr at HD initiation (Day)	Day of steroid initiation	Day of avacopan initiation	Prednisone (mg) at avacopan initiation	Remission (BVAS = 0)	Resolution of hematuria (Day)	Serologic remission (Day)	Day of last HD	Cr at last follow-up (Day)
1	2.3	4.9 (10)	4	15	40	Yes	155	Y (113)	125	2.2 (265)
2	5.54	5.6 (2)	2	49	15	Yes	150	Y (68)	192	2.0 (224)
3	4.8	6.9 (4)	1	9	60	Yes	124	Y (60)	14	1.4 (269)

BVAS, Birmingham Vasculitis Activity Score. Cr, serum creatinine. HD, hemodialysis.

Resolution of hematuria was defined as heme negativity on dipstick and ≤ 2 RBCs/hpf. Serologic remission was defined as a negative test for antibodies to myeloperoxidase or proteinase-3.

### Case 2

A 64-year-old woman with a history of hypertension presented to the hospital with progressive fatigue and dyspnea. The patient had no history of kidney disease and was found to have a serum creatinine of 5.5 mg/dl. Computed tomography of the chest revealed diffuse ground glass opacities. On hospital day 2, the patient developed hemoptysis and progressive hypoxemia requiring intubation. Bronchoalveolar lavage was consistent with diffuse alveolar hemorrhage. Hemodialysis was also initiated on hospital day 2 because of progressive acute kidney injury and severe metabolic acidosis. Pulse glucocorticoids were commenced empirically because of the high concern for a pulmonary-renal syndrome. Myeloperoxidase-ANCA returned elevated at 228.5 units (reference <1). Renal biopsy revealed cellular (*n* = 4) or fibrocellular crescents (*n* = 6) in all 10 glomeruli (mixed class,S2 Figure 1b). A severe, mononuclear-predominate interstitial infiltrate involved approximately 80% of the cortex, precluding accurate assessment of interstitial fibrosis and tubular atrophy.

Treatment consisted of pulse methylprednisolone for 1 week followed by a prednisone taper (Supplementary Table S1), plasma exchange (6 sessions), rituximab (2 doses of 1000 mg administered 2 weeks apart), and 1 pulse of 500 mg i.v. cyclophosphamide. Pulse methylprednisolone was administered as follows: 500 mg i.v. daily for 3 days, followed by 60 mg i.v. twice daily for 4 days. The patient had clinical resolution of diffuse alveolar hemorrhage and was discharged 31 days after presentation on hemodialysis. Because of difficulties in obtaining the medication as an inpatient, avacopan 30 mg twice daily was added as an outpatient 48 days after presentation to the hospital. Dialysis was discontinued on day 192 and the patient is in clinical remission with a serum creatinine of 2.0 mg/dl after 224 days of follow-up (Table 1). The patient remains on avacopan and was transitioned to maintenance therapy with rituximab.

### Case 3

A 72-year-old woman with a history of hypertension and hyperlipidemia presented to the hospital with progressive fatigue, epistaxis, and gross hematuria. Serum creatinine on presentation was elevated at 4.8 mg/dl with a urine albumin: creatinine ratio of 5383 mg/g and red blood cell casts on sediment. She had no prior history of renal disease and was a nonsmoker. Computed tomography of the chest revealed multiple scattered pulmonary nodules in both lungs. Intravenous pulse methylprednisolone was initiated on hospital day 1 given the clinical concern for rapidly progressive GN. Kidney function worsened, and hemodialysis was started on hospital day 4 (Table 1).

Workup was notable for dual positivity of anti-glomerular basement membrane (anti-GBM) antibodies and proteinase-3-ANCA. Renal biopsy demonstrated diffuse necrotizing and crescentic GN with cellular crescents involving 15 of 21 viable glomeruli (Figure 1c). Minimal chronicity was present with 5% global glomerulosclerosis and approximately 5% interstitial fibrosis and tubular atrophy. Immunofluorescence revealed 3+ linear global glomerular capillary wall staining for IgG consistent with anti-GBM nephritis (Figure 1d). Treatment consisted of methylprednisolone 500 mg i.v. daily for 3 days followed by a prednisone taper (Supplementary Table S1), 8 weeks of oral cyclophosphamide at a dose of 75 mg daily, and 9 sessions of plasma exchange. Avacopan 30 mg twice daily was added on hospital day 9. Hemodialysis was discontinued on hospital day 14 and the patient was discharged on day 16 with a serum creatinine of 4.6. Given the presence of ANCA, maintenance therapy with rituximab was initiated 3 months after presentation. Repeat chest computed tomography revealed resolution of the pulmonary nodules. The patient remains on avacopan with a creatinine of 1.4 mg/dl after 269 days of follow-up.

## Discussion

Blockade of the C5a receptor with avacopan during induction of remission therapy appears to improve kidney recovery in patients with ANCA GN.^5^ There are limited data, however, on using avacopan in patients with ANCA GN requiring KRT, with only 1 patient reported in the literature to date.S3 Patients with AAV requiring dialysis at presentation often have poor outcomes, with 40% to 50% of patients developing end-stage kidney disease or dying in the ensuing 6 months (Table 2).^3^^,^^6^ This understudied subgroup may derive the greatest relative benefit from treatment with avacopan. We present 3 consecutive patients with rapidly progressive GN requiring KRT treated with avacopan in combination with rituximab and/or cyclophosphamide and a rapid steroid taper, all of whom recovered sufficient kidney function to discontinue hemodialysis. One patient had concomitant anti-GBM antibodies, representing the first such patient, to the best of our knowledge, treated with avacopan. Hepatotoxicity is a potential side effect of avacopan, and monthly monitoring of liver function tests is recommended for the first 6 months of therapy. The medication was well tolerated in the 3 patients with no treatment-related adverse events, including no abnormalities in liver function tests. The 2 patients discharged on dialysis received care at local units, and it is possible that delayed recognition of kidney recovery led to a longer duration of dialysis than required.

**Table 2:** Teaching points

Teaching points
1. Morbidity and mortality in AAV are largely driven by kidney-related outcomes.
2. Alternative complement pathway activation leading to C5a generation plays a key role in driving the inflammatory cascade in AAV.
3. The use of avacopan during induction of remission therapy for AAV can reduce glucocorticoid exposure and may improve long-term kidney outcomes
4. The optimal glucocorticoid regimen to be used in conjunction with avacopan remains unknown.

Preexisting data from both animal and human studies suggest that blockade of the C5a receptor in ANCA GN has the potential to improve kidney outcomes. In a murine model expressing the human C5a receptor, treatment with avacopan was able to abrogate the development of pauci-immune necrotizing and crescentic GN following passive transfer of antibodies to mouse myeloperoxidase.S4 In addition, staining for C5b-9 on renal biopsies is positive in most patients with ANCA GN, confirming that complement activation occurs in human disease.S5 In the ADVOCATE trial, patients randomized to avacopan had more rapid reductions in albuminuria and greater recovery of eGFR despite a significant reduction in glucocorticoid exposure. Among patients entering ADVOCATE with an eGFR <30 ml/min per 1.73 m^2^, the least-squares mean increase in eGFR after 52 weeks was 13.7 and 5.6 ml/min per 1.73 m^2^ in the avacopan and prednisone groups, respectively. Together, these findings likely reflect a rapid attenuation of glomerular inflammation, which could translate into a reduction in irreversible kidney damage. There is also evidence that complement activation plays an important role in the pathogenesis of anti-GBM disease. On biopsies of patients with anti-GBM disease, immunofluorescence typically reveals C3 deposited with IgG along the glomerular capillary wall.^7^ Moreover, patients with anti-GBM disease have elevated levels of C5a and soluble C5b-9 in both plasma and urine.^8^ The role of complement blockade in the treatment of anti-GBM disease merits further investigation.

Avacopan is highly protein-bound (>99.9%) and lipophilic with an apparent volume of distribution of 345 l.S6 Although empiric data are lacking, the pharmacokinetic data suggest that hemodialysis would have little impact on plasma drug levels, therefore obviating the need for dose adjustment. All 3 patients in this series were also treated with plasma exchange. Whereas plasma exchange would be expected to remove a large portion of avacopan in circulation, this fraction only accounts for a minority of total drug distributed throughout the body. Avacopan was administered after plasma exchange, but the dose was not increased with the expectation that drug reequilibration from tissues into blood coupled with ongoing dosing would maintain drug levels close to the target concentration. Additional studies are required to formally investigate the effects of hemodialysis and plasma exchange on avacopan pharmacokinetics.

The optimal dosing of glucocorticoids in conjunction with avacopan is unknown. In the ADVOCATE trial, patients in the placebo group received a 20-week glucocorticoid taper similar to the lower-dose regimen used in the PEXIVAS trial.^5^^,^^9^ Patients randomized to avacopan did not receive study-supplied prednisone. However, at the discretion of the investigator, patients in both arms could receive non-study–supplied prednisone at an initial dose of ≤20 mg at study initiation with a required taper to discontinuation by week 4. Given the absence of data in patients with ANCA GN requiring KRT, a slightly more aggressive glucocorticoid taper was used in the 2 patients with AAV alone (Supplementary Table S1). Furthermore, a more prolonged taper was used in the patient with anti-GBM antibodies given the lack of evidence that C5a blockade can facilitate rapid steroid tapering in this population. Further investigation is required to inform glucocorticoid use in patients with AAV receiving avacopan. In particular, whether patients with severe kidney disease benefit from glucocorticoids in addition to avacopan remains unknown.

Another area of uncertainty is the optimal duration of avacopan therapy. We plan to continue avacopan for 1 year in these patients, consistent with the regimen used in the ADVOCATE trial. It is possible, however, that certain patient subgroups may benefit from a more prolonged course of avacopan, whereas a shorter course may be sufficient in others.

AAV patients with severe GN requiring KRT are often excluded from clinical trials. Treatment with avacopan in our patients appeared effective and was well tolerated, indicating C5a blockade may provide an incremental benefit in the management of this important high-risk group. Moreover, the response of the patient with anti-GBM disease raises the possibility that avacopan may also improve outcomes in this aggressive disease process. Given the small number of patients, the results presented are only hypothesis-generating. Additional studies with comparator groups are needed to optimally define the benefit of C5a blockade in these populations.

## Disclosure

FBC has served as a consultant for ChemoCentryx, Valenza Bio, Travere Therapeutics, and Aurinia Pharmaceuticals, and has received speaking fees from ChemoCentryx, Aurinia Pharmaceuticals, and Calliditas Therapeutics. All the other authors declared no competing interests.

## Patient Consent

Consent was obtained from each patient to be included in this report.

## Supplementary Material

Supplementary File (PDF)

**Table S1.** Prednisone taper administered to patients.

**Supplementary References.**
