# Supplementary material for: Avacopan in Patients With Rapidly Progressive Glomerulonephritis Requiring Dialysis
Source: Kidney Int Rep. 2023 May 29;8(8):1687–91. doi: 10.1016/j.ekir.2023.05.017 (PMC10403643; doi:10.1016/j.ekir.2023.05.017)
Supplement: Supplementary File (PDF) [file mmc1.pdf]

Supplementary Material

Week	Prednisone (mg) AAV	Prednisone (mg) anti-GBM + AAV
1	60 mg	60 mg
2	40 mg	40 mg
3	30 mg	30 mg
4	20 mg	20 mg
5-6	15 mg	15 mg
7-8	10 mg	15 mg
9-10	5 mg	10 mg
11-12	0	10 mg
13-14	0	5 mg
15-16	0	5 mg

Supplemental Table. Prednisone taper administered to patients. AAV, anti-neutrophil cytoplasmic antibody associated vasculitis. Anti-GBM, anti-glomerular basement membrane disease

Supplemental References

S1. Walsh M, Collister D, Zeng L, *et al.* The effects of plasma exchange in patients with ANCA-associated vasculitis: an updated systematic review and meta-analysis. *BMJ* 2022; **376:** e064604.

S2. Berden AE, Ferrario F, Hagen EC, *et al.* Histopathologic classification of ANCA-associated glomerulonephritis. *J Am Soc Nephrol* 2010; **21:** 1628-1636.

S3. Gabilan C, Pfirmann P, Ribes D, *et al.* Avacopan as First-Line Treatment in Antineutrophil Cytoplasmic Antibody-Associated Vasculitis: A Steroid-Sparing Option. *Kidney Int Rep* 2022; **7:** 1115-1118.

S4. Xiao H, Dairaghi DJ, Powers JP, *et al.* C5a receptor (CD88) blockade protects against MPO-ANCA GN. *J Am Soc Nephrol* 2014; **25:** 225-231.

S5. Hilhorst M, van Paassen P, van Rie H, *et al.* Complement in ANCA-associated glomerulonephritis. *Nephrol Dial Transplant* 2017; **32:** 1302-1313.

S6. National Center for Biotechnology Information. PubChem Compound Summary for CID 49841217, Avacopan. https://pubchem.ncbi.nlm.nih.gov/compound/Avacopan. Accessed Nov. 12, 2022.
